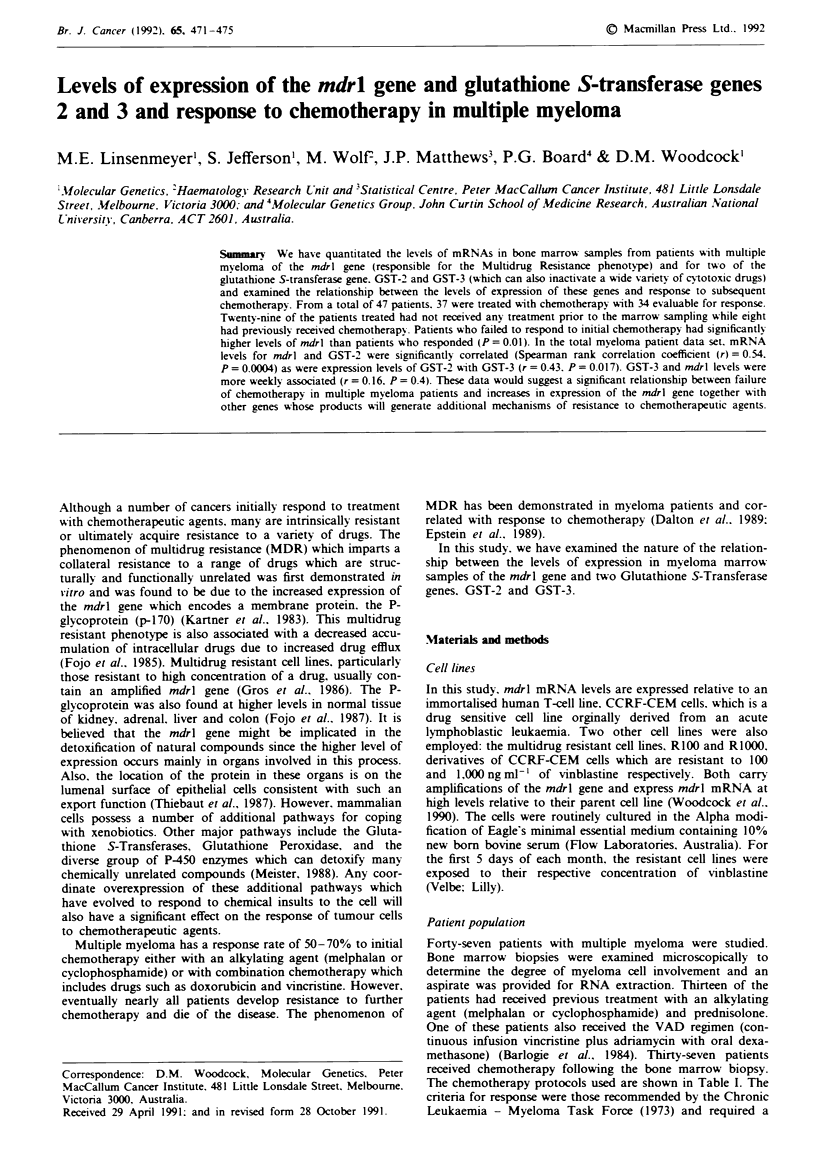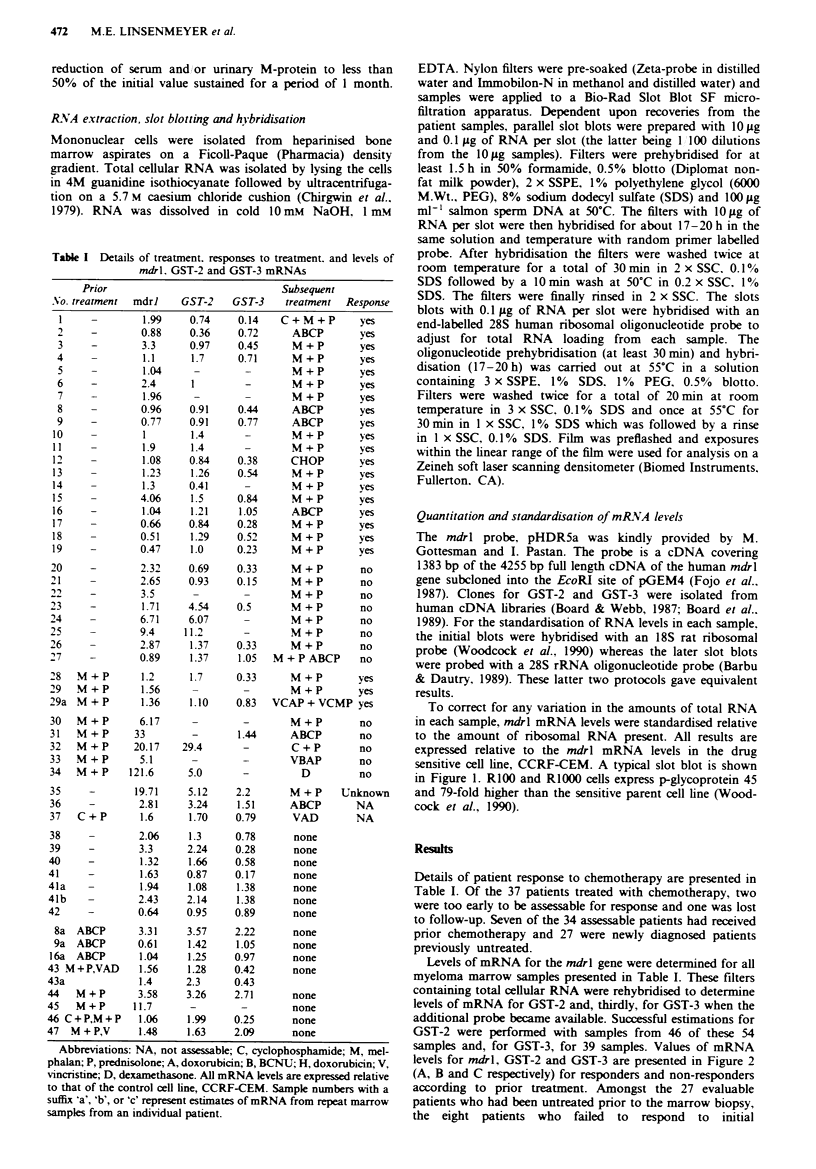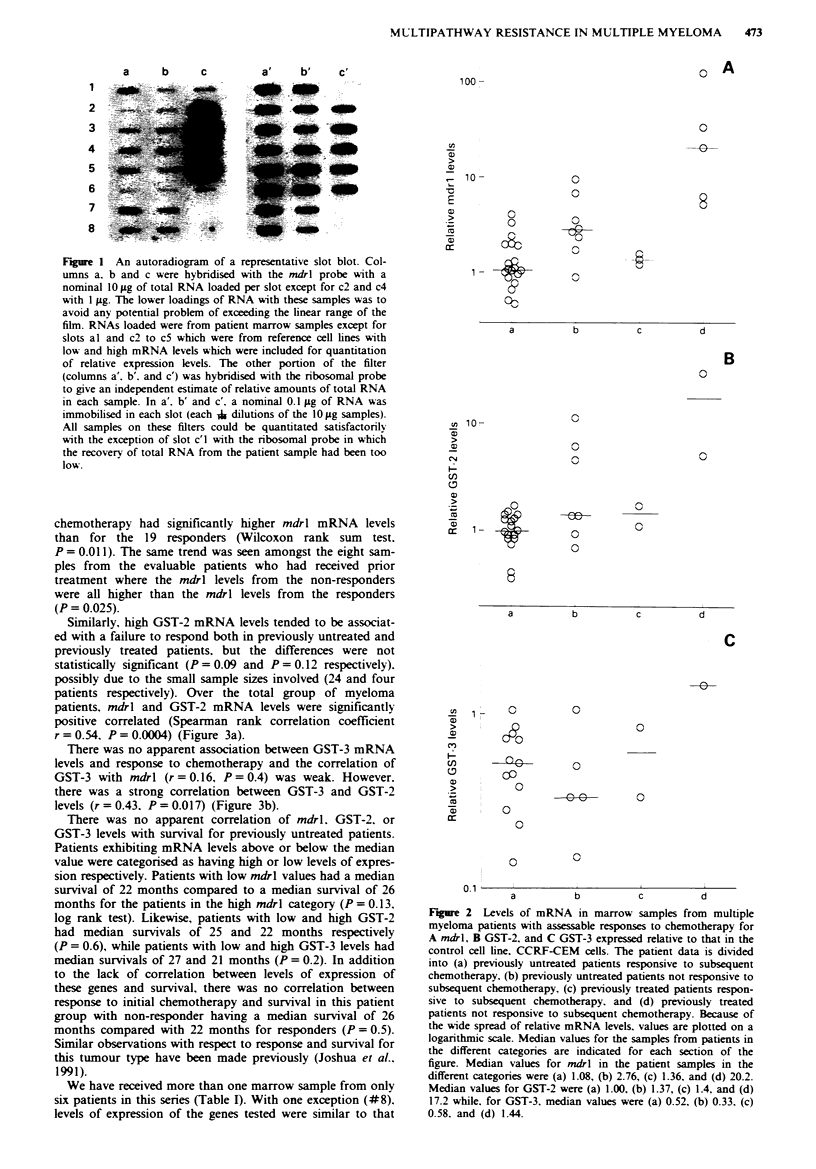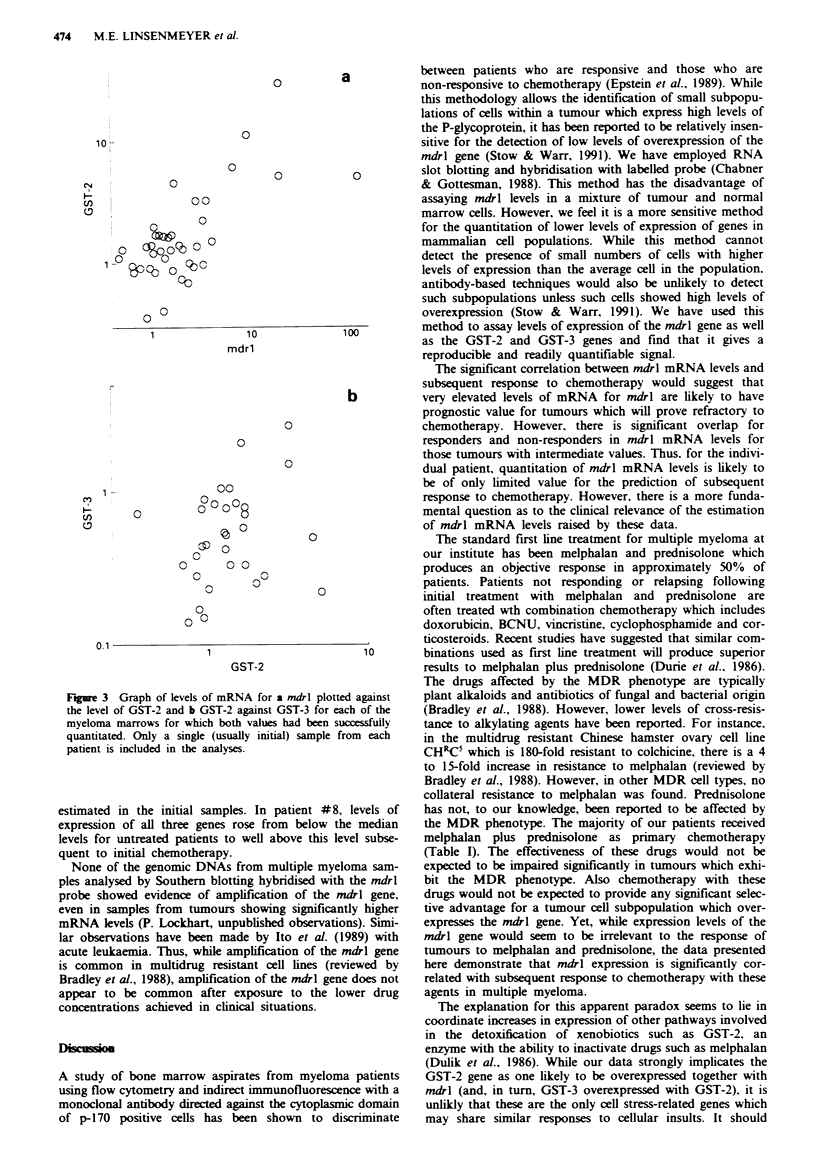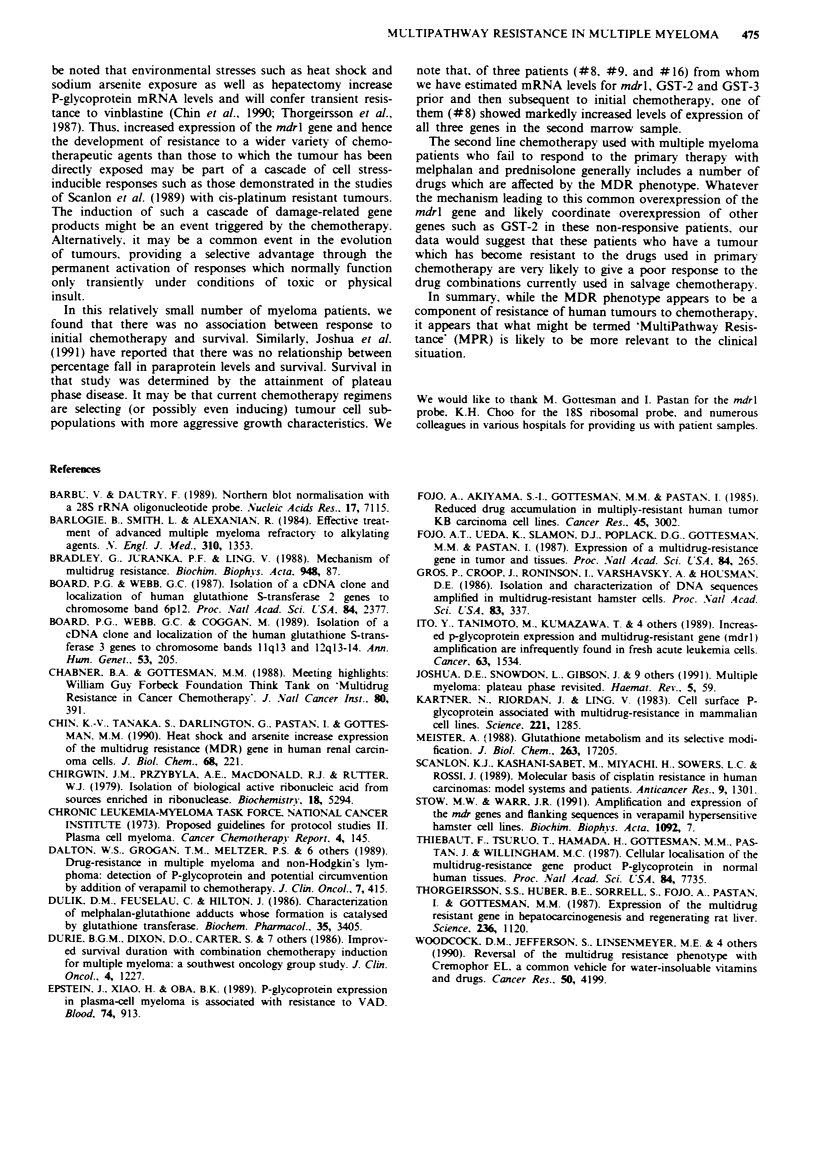# Levels of expression of the mdr1 gene and glutathione S-transferase genes 2 and 3 and response to chemotherapy in multiple myeloma.

**DOI:** 10.1038/bjc.1992.95

**Published:** 1992-03

**Authors:** M. E. Linsenmeyer, S. Jefferson, M. Wolf, J. P. Matthews, P. G. Board, D. M. Woodcock

**Affiliations:** Molecular Genetics, Peter MacCallum Cancer Institute, Melbourne, Victoria, Australia.

## Abstract

**Images:**


Br. J. Cancer (1992), 65, 471-475

© Macmillan Press Ltd., 1992

### Levels of expression of the *mdr*1 gene and glutathione *S*-transferase genes 2 and 3 and response to chemotherapy in multiple myeloma

M.E. Linsenmeyer[1], S. Jefferson[1], M. Wolf[2], J.P. Matthews[3], P.G. Board[4] & D.M. Woodcock[1]

[1]*Molecular Genetics,* [2]*Haematology Research Unit and* [3]*Statistical Centre, Peter MacCallum Cancer Institute, 481 Little Lonsdale Street, Melbourne, Victoria 3000; and* [4]*Molecular Genetics Group, John Curtin School of Medicine Research, Australian National University, Canberra, ACT 2601, Australia.*

**Summary** We have quantitated the levels of mRNAs in bone marrow samples from patients with multiple myeloma of the *mdr*1 gene (responsible for the Multidrug Resistance phenotype) and for two of the glutathione *S*-transferase gene, GST-2 and GST-3 (which can also inactivate a wide variety of cytotoxic drugs) and examined the relationship between the levels of expression of these genes and response to subsequent chemotherapy. From a total of 47 patients, 37 were treated with chemotherapy with 34 evaluable for response. Twenty-nine of the patients treated had not received any treatment prior to the marrow sampling while eight had previously received chemotherapy. Patients who failed to respond to initial chemotherapy had significantly higher levels of *mdr*1 than patients who responded ($P = 0.01$). In the total myeloma patient data set, mRNA levels for *mdr*1 and GST-2 were significantly correlated (Spearman rank correlation coefficient ($r$) = 0.54, $P = 0.0004$) as were expression levels of GST-2 with GST-3 ($r = 0.43$, $P = 0.017$). GST-3 and *mdr*1 levels were more weekly associated ($r = 0.16$, $P = 0.4$). These data would suggest a significant relationship between failure of chemotherapy in multiple myeloma patients and increases in expression of the *mdr*1 gene together with other genes whose products will generate additional mechanisms of resistance to chemotherapeutic agents.

Although a number of cancers initially respond to treatment with chemotherapeutic agents, many are intrinsically resistant or ultimately acquire resistance to a variety of drugs. The phenomenon of multidrug resistance (MDR) which imparts a collateral resistance to a range of drugs which are structurally and functionally unrelated was first demonstrated *in vitro* and was found to be due to the increased expression of the *mdr*1 gene which encodes a membrane protein, the P-glycoprotein (p-170) (Kartner *et al.*, 1983). This multidrug resistant phenotype is also associated with a decreased accumulation of intracellular drugs due to increased drug efflux (Fojo *et al.*, 1985). Multidrug resistant cell lines, particularly those resistant to high concentration of a drug, usually contain an amplified *mdr*1 gene (Gros *et al.*, 1986). The P-glycoprotein was also found at higher levels in normal tissue of kidney, adrenal, liver and colon (Fojo *et al.*, 1987). It is believed that the *mdr*1 gene might be implicated in the detoxification of natural compounds since the higher level of expression occurs mainly in organs involved in this process. Also, the location of the protein in these organs is on the lumenal surface of epithelial cells consistent with such an export function (Thiebaut *et al.*, 1987). However, mammalian cells possess a number of additional pathways for coping with xenobiotics. Other major pathways include the Glutathione *S*-Transferases, Glutathione Peroxidase, and the diverse group of P-450 enzymes which can detoxify many chemically unrelated compounds (Meister, 1988). Any coordinate overexpression of these additional pathways which have evolved to respond to chemical insults to the cell will also have a significant effect on the response of tumour cells to chemotherapeutic agents.

Multiple myeloma has a response rate of 50-70% to initial chemotherapy either with an alkylating agent (melphalan or cyclophosphamide) or with combination chemotherapy which includes drugs such as doxorubicin and vincristine. However, eventually nearly all patients develop resistance to further chemotherapy and die of the disease. The phenomenon of MDR has been demonstrated in myeloma patients and correlated with response to chemotherapy (Dalton *et al.*, 1989; Epstein *et al.*, 1989).

In this study, we have examined the nature of the relationship between the levels of expression in myeloma marrow samples of the *mdr*1 gene and two Glutathione *S*-Transferase genes, GST-2 and GST-3.

### Materials and methods

### Cell lines

In this study, *mdr*1 mRNA levels are expressed relative to an immortalised human T-cell line, CCRF-CEM cells, which is a drug sensitive cell line orginally derived from an acute lymphoblastic leukaemia. Two other cell lines were also employed: the multidrug resistant cell lines, R100 and R1000, derivatives of CCRF-CEM cells which are resistant to 100 and 1,000 ng ml$^{-1}$ of vinblastine respectively. Both carry amplifications of the *mdr*1 gene and express *mdr*1 mRNA at high levels relative to their parent cell line (Woodcock *et al.*, 1990). The cells were routinely cultured in the Alpha modification of Eagle's minimal essential medium containing 10% new born bovine serum (Flow Laboratories, Australia). For the first 5 days of each month, the resistant cell lines were exposed to their respective concentration of vinblastine (Velbe; Lilly).

### Patient population

Forty-seven patients with multiple myeloma were studied. Bone marrow biopsies were examined microscopically to determine the degree of myeloma cell involvement and an aspirate was provided for RNA extraction. Thirteen of the patients had received previous treatment with an alkylating agent (melphalan or cyclophosphamide) and prednisolone. One of these patients also received the VAD regimen (continuous infusion vincristine plus adriamycin with oral dexamethasone) (Barlogie *et al.*, 1984). Thirty-seven patients received chemotherapy following the bone marrow biopsy. The chemotherapy protocols used are shown in Table I. The criteria for response were those recommended by the Chronic Leukaemia - Myeloma Task Force (1973) and required a

Correspondence: D.M. Woodcock, Molecular Genetics, Peter MacCallum Cancer Institute, 481 Little Lonsdale Street, Melbourne, Victoria 3000, Australia.

Received 29 April 1991; and in revised form 28 October 1991.

reduction of serum and/or urinary M-protein to less than 50% of the initial value sustained for a period of 1 month.

*RNA extraction, slot blotting and hybridisation*

Mononuclear cells were isolated from heparinised bone marrow aspirates on a Ficoll-Paque (Pharmacia) density gradient. Total cellular RNA was isolated by lysing the cells in 4M guanidine isothiocyanate followed by ultracentrifugation on a 5.7 M caesium chloride cushion (Chirgwin *et al.*, 1979). RNA was dissolved in cold 10 mM NaOH, 1 mM EDTA. Nylon filters were pre-soaked (Zeta-probe in distilled water and Immobilon-N in methanol and distilled water) and samples were applied to a Bio-Rad Slot Blot SF microfiltration apparatus. Dependent upon recoveries from the patient samples, parallel slot blots were prepared with 10 μg and 0.1 μg of RNA per slot (the latter being 1/100 dilutions from the 10 μg samples). Filters were prehybridised for at least 1.5 h in 50% formamide, 0.5% blotto (Diplomat non-fat milk powder), 2 × SSPE, 1% polyethylene glycol (6000 M.Wt., PEG), 8% sodium dodecyl sulfate (SDS) and 100 μg $ml^{-1}$ salmon sperm DNA at 50°C. The filters with 10 μg of RNA per slot were then hybridised for about 17–20 h in the same solution and temperature with random primer labelled probe. After hybridisation the filters were washed twice at room temperature for a total of 30 min in 2 × SSC, 0.1% SDS followed by a 10 min wash at 50°C in 0.2 × SSC, 1% SDS. The filters were finally rinsed in 2 × SSC. The slots blots with 0.1 μg of RNA per slot were hybridised with an end-labelled 28S human ribosomal oligonucleotide probe to adjust for total RNA loading from each sample. The oligonucleotide prehybridisation (at least 30 min) and hybridisation (17–20 h) was carried out at 55°C in a solution containing 3 × SSPE, 1% SDS, 1% PEG, 0.5% blotto. Filters were washed twice for a total of 20 min at room temperature in 3 × SSC, 0.1% SDS and once at 55°C for 30 min in 1 × SSC, 1% SDS which was followed by a rinse in 1 × SSC, 0.1% SDS. Film was preflashed and exposures within the linear range of the film were used for analysis on a Zeineh soft laser scanning densitometer (Biomed Instruments, Fullerton, CA).

**Table I** Details of treatment, responses to treatment, and levels of *mdr*1, GST-2 and GST-3 mRNAs

| No. | Prior treatment | mdr*1* | *GST-2* | *GST-3* | Subsequent treatment | Response |
|---|---|---|---|---|---|---|
| 1 | – | 1.99 | 0.74 | 0.14 | C + M + P | yes |
| 2 | – | 0.88 | 0.36 | 0.72 | ABCP | yes |
| 3 | – | 3.3 | 0.97 | 0.45 | M + P | yes |
| 4 | – | 1.1 | 1.7 | 0.71 | M + P | yes |
| 5 | – | 1.04 | – | – | M + P | yes |
| 6 | – | 2.4 | 1 | – | M + P | yes |
| 7 | – | 1.96 | – | – | M + P | yes |
| 8 | – | 0.96 | 0.91 | 0.44 | ABCP | yes |
| 9 | – | 0.77 | 0.91 | 0.77 | ABCP | yes |
| 10 | – | 1 | 1.4 | – | M + P | yes |
| 11 | – | 1.9 | 1.4 | – | M + P | yes |
| 12 | – | 1.08 | 0.84 | 0.38 | CHOP | yes |
| 13 | – | 1.23 | 1.26 | 0.54 | M + P | yes |
| 14 | – | 1.3 | 0.41 | – | M + P | yes |
| 15 | – | 4.06 | 1.5 | 0.84 | M + P | yes |
| 16 | – | 1.04 | 1.21 | 1.05 | ABCP | yes |
| 17 | – | 0.66 | 0.84 | 0.28 | M + P | yes |
| 18 | – | 0.51 | 1.29 | 0.52 | M + P | yes |
| 19 | – | 0.47 | 1.0 | 0.23 | M + P | yes |
| 20 | – | 2.32 | 0.69 | 0.33 | M + P | no |
| 21 | – | 2.65 | 0.93 | 0.15 | M + P | no |
| 22 | – | 3.5 | – | – | M + P | no |
| 23 | – | 1.71 | 4.54 | 0.5 | M + P | no |
| 24 | – | 6.71 | 6.07 | – | M + P | no |
| 25 | – | 9.4 | 11.2 | – | M + P | no |
| 26 | – | 2.87 | 1.37 | 0.33 | M + P | no |
| 27 | – | 0.89 | 1.37 | 1.05 | M + P ABCP | no |
| 28 | M + P | 1.2 | 1.7 | 0.33 | M + P | yes |
| 29 | M + P | 1.56 | – | – | M + P | yes |
| 29a | M + P | 1.36 | 1.10 | 0.83 | VCAP + VCMP | yes |
| 30 | M + P | 6.17 | – | – | M + P | no |
| 31 | M + P | 33 | – | 1.44 | ABCP | no |
| 32 | M + P | 20.17 | 29.4 | – | C + P | no |
| 33 | M + P | 5.1 | – | – | VBAP | no |
| 34 | M + P | 121.6 | 5.0 | – | D | no |
| 35 | – | 19.71 | 5.12 | 2.2 | M + P | Unknown |
| 36 | – | 2.81 | 3.24 | 1.51 | ABCP | NA |
| 37 | C + P | 1.6 | 1.70 | 0.79 | VAD | NA |
| 38 | – | 2.06 | 1.3 | 0.78 | none | |
| 39 | – | 3.3 | 2.24 | 0.28 | none | |
| 40 | – | 1.32 | 1.66 | 0.58 | none | |
| 41 | – | 1.63 | 0.87 | 0.17 | none | |
| 41a | – | 1.94 | 1.08 | 1.38 | none | |
| 41b | – | 2.43 | 2.14 | 1.38 | none | |
| 42 | – | 0.64 | 0.95 | 0.89 | none | |
| 8a | ABCP | 3.31 | 3.57 | 2.22 | none | |
| 9a | ABCP | 0.61 | 1.42 | 1.05 | none | |
| 16a | ABCP | 1.04 | 1.25 | 0.97 | none | |
| 43 | M+P,VAD | 1.56 | 1.28 | 0.42 | none | |
| 43a | | 1.4 | 2.3 | 0.43 | | |
| 44 | M + P | 3.58 | 3.26 | 2.71 | none | |
| 45 | M + P | 11.7 | – | – | none | |
| 46 | C+P,M+P | 1.06 | 1.99 | 0.25 | none | |
| 47 | M + P,V | 1.48 | 1.63 | 2.09 | none | |

Abbreviations: NA, not assessable; C, cyclophosphamide; M, melphalan; P, prednisolone; A, doxorubicin; B, BCNU; H, doxorubicin; V, vincristine; D, dexamethasone. All mRNA levels are expressed relative to that of the control cell line, CCRF-CEM. Sample numbers with a suffix 'a', 'b', or 'c' represent estimates of mRNA from repeat marrow samples from an individual patient.

*Quantitation and standardisation of mRNA levels*

The *mdr*1 probe, pHDR5a was kindly provided by M. Gottesman and I. Pastan. The probe is a cDNA covering 1383 bp of the 4255 bp full length cDNA of the human *mdr*1 gene subcloned into the *Eco*RI site of pGEM4 (Fojo *et al.*, 1987). Clones for GST-2 and GST-3 were isolated from human cDNA libraries (Board & Webb, 1987; Board *et al.*, 1989). For the standardisation of RNA levels in each sample, the initial blots were hybridised with an 18S rat ribosomal probe (Woodcock *et al.*, 1990) whereas the later slot blots were probed with a 28S rRNA oligonucleotide probe (Barbu & Dautry, 1989). These latter two protocols gave equivalent results.

To correct for any variation in the amounts of total RNA in each sample, *mdr*1 mRNA levels were standardised relative to the amount of ribosomal RNA present. All results are expressed relative to the *mdr*1 mRNA levels in the drug sensitive cell line, CCRF-CEM. A typical slot blot is shown in Figure 1. R100 and R1000 cells express p-glycoprotein 45 and 79-fold higher than the sensitive parent cell line (Woodcock *et al.*, 1990).

### Results

Details of patient response to chemotherapy are presented in Table I. Of the 37 patients treated with chemotherapy, two were too early to be assessable for response and one was lost to follow-up. Seven of the 34 assessable patients had received prior chemotherapy and 27 were newly diagnosed patients previously untreated.

Levels of mRNA for the *mdr*1 gene were determined for all myeloma marrow samples presented in Table I. These filters containing total cellular RNA were rehybridised to determine levels of mRNA for GST-2 and, thirdly, for GST-3 when the additional probe became available. Successful estimations for GST-2 were performed with samples from 46 of these 54 samples and, for GST-3, for 39 samples. Values of mRNA levels for *mdr*1, GST-2 and GST-3 are presented in Figure 2 (A, B and C respectively) for responders and non-responders according to prior treatment. Amongst the 27 evaluable patients who had been untreated prior to the marrow biopsy, the eight patients who failed to respond to initial

**Figure 1** An autoradiogram of a representative slot blot. Columns a, b and c were hybridised with the *mdr*l probe with a nominal 10 μg of total RNA loaded per slot except for c2 and c4 with 1 μg. The lower loadings of RNA with these samples was to avoid any potential problem of exceeding the linear range of the film. RNAs loaded were from patient marrow samples except for slots a1 and c2 to c5 which were from reference cell lines with low and high mRNA levels which were included for quantitation of relative expression levels. The other portion of the filter (columns a', b', and c') was hybridised with the ribosomal probe to give an independent estimate of relative amounts of total RNA in each sample. In a', b' and c', a nominal 0.1 μg of RNA was immobilised in each slot (each $\frac{1}{100}$ dilutions of the 10 μg samples). All samples on these filters could be quantitated satisfactorily with the exception of slot c'1 with the ribosomal probe in which the recovery of total RNA from the patient sample had been too low.

chemotherapy had significantly higher *mdr*l mRNA levels than for the 19 responders (Wilcoxon rank sum test, $P = 0.011$). The same trend was seen amongst the eight samples from the evaluable patients who had received prior treatment where the *mdr*l levels from the non-responders were all higher than the *mdr*l levels from the responders ($P = 0.025$).

Similarly, high GST-2 mRNA levels tended to be associated with a failure to respond both in previously untreated and previously treated patients, but the differences were not statistically significant ($P = 0.09$ and $P = 0.12$ respectively), possibly due to the small sample sizes involved (24 and four patients respectively). Over the total group of myeloma patients, *mdr*l and GST-2 mRNA levels were significantly positive correlated (Spearman rank correlation coefficient $r = 0.54$, $P = 0.0004$) (Figure 3a).

There was no apparent association between GST-3 mRNA levels and response to chemotherapy and the correlation of GST-3 with *mdr*l ($r = 0.16$, $P = 0.4$) was weak. However, there was a strong correlation between GST-3 and GST-2 levels ($r = 0.43$, $P = 0.017$) (Figure 3b).

There was no apparent correlation of *mdr*l, GST-2, or GST-3 levels with survival for previously untreated patients. Patients exhibiting mRNA levels above or below the median value were categorised as having high or low levels of expression respectively. Patients with low *mdr*l values had a median survival of 22 months compared to a median survival of 26 months for the patients in the high *mdr*l category ($P = 0.13$, log rank test). Likewise, patients with low and high GST-2 had median survivals of 25 and 22 months respectively ($P = 0.6$), while patients with low and high GST-3 levels had median survivals of 27 and 21 months ($P = 0.2$). In addition to the lack of correlation between levels of expression of these genes and survival, there was no correlation between response to initial chemotherapy and survival in this patient group with non-responder having a median survival of 26 months compared with 22 months for responders ($P = 0.5$). Similar observations with respect to response and survival for this tumour type have been made previously (Joshua *et al.*, 1991).

We have received more than one marrow sample from only six patients in this series (Table I). With one exception (#8), levels of expression of the genes tested were similar to that

**Figure 2** Levels of mRNA in marrow samples from multiple myeloma patients with assessable responses to chemotherapy for **A** *mdr*l, **B** GST-2, and **C** GST-3 expressed relative to that in the control cell line, CCRF-CEM cells. The patient data is divided into (a) previously untreated patients responsive to subsequent chemotherapy, (b) previously untreated patients not responsive to subsequent chemotherapy, (c) previously treated patients responsive to subsequent chemotherapy, and (d) previously treated patients not responsive to subsequent chemotherapy. Because of the wide spread of relative mRNA levels, values are plotted on a logarithmic scale. Median values for the samples from patients in the different categories are indicated for each section of the figure. Median values for *mdr*l in the patient samples in the different categories were (a) 1.08, (b) 2.76, (c) 1.36, and (d) 20.2. Median values for GST-2 were (a) 1.00, (b) 1.37, (c) 1.4, and (d) 17.2 while, for GST-3, median values were (a) 0.52, (b) 0.33, (c) 0.58, and (d) 1.44.

**Figure 3** Graph of levels of mRNA for **a** *mdr*1 plotted against the level of GST-2 and **b** GST-2 against GST-3 for each of the myeloma marrows for which both values had been successfully quantitated. Only a single (usually initial) sample from each patient is included in the analyses.

estimated in the initial samples. In patient #8, levels of expression of all three genes rose from below the median levels for untreated patients to well above this level subsequent to initial chemotherapy.

None of the genomic DNAs from multiple myeloma samples analysed by Southern blotting hybridised with the *mdr*1 probe showed evidence of amplification of the *mdr*1 gene, even in samples from tumours showing significantly higher mRNA levels (P. Lockhart, unpublished observations). Similar observations have been made by Ito *et al.* (1989) with acute leukaemia. Thus, while amplification of the *mdr*1 gene is common in multidrug resistant cell lines (reviewed by Bradley *et al.*, 1988), amplification of the *mdr*1 gene does not appear to be common after exposure to the lower drug concentrations achieved in clinical situations.

### Discussion

A study of bone marrow aspirates from myeloma patients using flow cytometry and indirect immunofluorescence with a monoclonal antibody directed against the cytoplasmic domain of p-170 positive cells has been shown to discriminate between patients who are responsive and those who are non-responsive to chemotherapy (Epstein *et al.*, 1989). While this methodology allows the identification of small subpopulations of cells within a tumour which express high levels of the P-glycoprotein, it has been reported to be relatively insensitive for the detection of low levels of overexpression of the *mdr*1 gene (Stow & Warr, 1991). We have employed RNA slot blotting and hybridisation with labelled probe (Chabner & Gottesman, 1988). This method has the disadvantage of assaying *mdr*1 levels in a mixture of tumour and normal marrow cells. However, we feel it is a more sensitive method for the quantitation of lower levels of expression of genes in mammalian cell populations. While this method cannot detect the presence of small numbers of cells with higher levels of expression than the average cell in the population, antibody-based techniques would also be unlikely to detect such subpopulations unless such cells showed high levels of overexpression (Stow & Warr, 1991). We have used this method to assay levels of expression of the *mdr*1 gene as well as the GST-2 and GST-3 genes and find that it gives a reproducible and readily quantifiable signal.

The significant correlation between *mdr*1 mRNA levels and subsequent response to chemotherapy would suggest that very elevated levels of mRNA for *mdr*1 are likely to have prognostic value for tumours which will prove refractory to chemotherapy. However, there is significant overlap for responders and non-responders in *mdr*1 mRNA levels for those tumours with intermediate values. Thus, for the individual patient, quantitation of *mdr*1 mRNA levels is likely to be of only limited value for the prediction of subsequent response to chemotherapy. However, there is a more fundamental question as to the clinical relevance of the estimation of *mdr*1 mRNA levels raised by these data.

The standard first line treatment for multiple myeloma at our institute has been melphalan and prednisolone which produces an objective response in approximately 50% of patients. Patients not responding or relapsing following initial treatment with melphalan and prednisolone are often treated wth combination chemotherapy which includes doxorubicin, BCNU, vincristine, cyclophosphamide and corticosteroids. Recent studies have suggested that similar combinations used as first line treatment will produce superior results to melphalan plus prednisolone (Durie *et al.*, 1986). The drugs affected by the MDR phenotype are typically plant alkaloids and antibiotics of fungal and bacterial origin (Bradley *et al.*, 1988). However, lower levels of cross-resistance to alkylating agents have been reported. For instance, in the multidrug resistant Chinese hamster ovary cell line $CH^RC^5$ which is 180-fold resistant to colchicine, there is a 4 to 15-fold increase in resistance to melphalan (reviewed by Bradley *et al.*, 1988). However, in other MDR cell types, no collateral resistance to melphalan was found. Prednisolone has not, to our knowledge, been reported to be affected by the MDR phenotype. The majority of our patients received melphalan plus prednisolone as primary chemotherapy (Table I). The effectiveness of these drugs would not be expected to be impaired significantly in tumours which exhibit the MDR phenotype. Also chemotherapy with these drugs would not be expected to provide any significant selective advantage for a tumour cell subpopulation which overexpresses the *mdr*1 gene. Yet, while expression levels of the *mdr*1 gene would seem to be irrelevant to the response of tumours to melphalan and prednisolone, the data presented here demonstrate that *mdr*1 expression is significantly correlated with subsequent response to chemotherapy with these agents in multiple myeloma.

The explanation for this apparent paradox seems to lie in coordinate increases in expression of other pathways involved in the detoxification of xenobiotics such as GST-2, an enzyme with the ability to inactivate drugs such as melphalan (Dulik *et al.*, 1986). While our data strongly implicates the GST-2 gene as one likely to be overexpressed together with *mdr*1 (and, in turn, GST-3 overexpressed with GST-2), it is unlikly that these are the only cell stress-related genes which may share similar responses to cellular insults. It should be noted that environmental stresses such as heat shock and sodium arsenite exposure as well as hepatectomy increase P-glycoprotein mRNA levels and will confer transient resistance to vinblastine (Chin *et al.*, 1990; Thorgeirsson *et al.*, 1987). Thus, increased expression of the *mdr*1 gene and hence the development of resistance to a wider variety of chemotherapeutic agents than those to which the tumour has been directly exposed may be part of a cascade of cell stress-inducible responses such as those demonstrated in the studies of Scanlon *et al.* (1989) with cis-platinum resistant tumours. The induction of such a cascade of damage-related gene products might be an event triggered by the chemotherapy. Alternatively, it may be a common event in the evolution of tumours, providing a selective advantage through the permanent activation of responses which normally function only transiently under conditions of toxic or physical insult.

In this relatively small number of myeloma patients, we found that there was no association between response to initial chemotherapy and survival. Similarly, Joshua *et al.* (1991) have reported that there was no relationship between percentage fall in paraprotein levels and survival. Survival in that study was determined by the attainment of plateau phase disease. It may be that current chemotherapy regimens are selecting (or possibly even inducing) tumour cell subpopulations with more aggressive growth characteristics. We note that, of three patients (#8, #9, and #16) from whom we have estimated mRNA levels for *mdr*1, GST-2 and GST-3 prior and then subsequent to initial chemotherapy, one of them (#8) showed markedly increased levels of expression of all three genes in the second marrow sample.

The second line chemotherapy used with multiple myeloma patients who fail to respond to the primary therapy with melphalan and prednisolone generally includes a number of drugs which are affected by the MDR phenotype. Whatever the mechanism leading to this common overexpression of the *mdr*1 gene and likely coordinate overexpression of other genes such as GST-2 in these non-responsive patients, our data would suggest that these patients who have a tumour which has become resistant to the drugs used in primary chemotherapy are very likely to give a poor response to the drug combinations currently used in salvage chemotherapy.

In summary, while the MDR phenotype appears to be a component of resistance of human tumours to chemotherapy, it appears that what might be termed 'MultiPathway Resistance' (MPR) is likely to be more relevant to the clinical situation.

We would like to thank M. Gottesman and I. Pastan for the *mdr*1 probe, K.H. Choo for the 18S ribosomal probe, and numerous colleagues in various hospitals for providing us with patient samples.

**References**

BARBU, V. & DAUTRY, F. (1989). Northern blot normalisation with a 28S rRNA oligonucleotide probe. *Nucleic Acids Res.*, **17**, 7115.

BARLOGIE, B., SMITH, L. & ALEXANIAN, R. (1984). Effective treatment of advanced multiple myeloma refractory to alkylating agents. *N. Engl. J. Med.*, **310**, 1353.

BRADLEY, G., JURANKA, P.F. & LING, V. (1988). Mechanism of multidrug resistance. *Biochim. Biophys. Acta*, **948**, 87.

BOARD, P.G. & WEBB, G.C. (1987). Isolation of a cDNA clone and localization of human glutathione S-transferase 2 genes to chromosome band 6p12. *Proc. Natl Acad. Sci. USA*, **84**, 2377.

BOARD, P.G., WEBB, G.C. & COGGAN, M. (1989). Isolation of a cDNA clone and localization of the human glutathione S-transferase 3 genes to chromosome bands 11q13 and 12q13-14. *Ann. Hum. Genet.*, **53**, 205.

CHABNER, B.A. & GOTTESMAN, M.M. (1988). Meeting highlights: William Guy Forbeck Foundation Think Tank on 'Multidrug Resistance in Cancer Chemotherapy'. *J. Natl Cancer Inst.*, **80**, 391.

CHIN, K.-V., TANAKA, S., DARLINGTON, G., PASTAN, I. & GOTTESMAN, M.M. (1990). Heat shock and arsenite increase expression of the multidrug resistance (MDR) gene in human renal carcinoma cells. *J. Biol. Chem.*, **68**, 221.

CHIRGWIN, J.M., PRZYBYLA, A.E., MACDONALD, R.J. & RUTTER, W.J. (1979). Isolation of biological active ribonucleic acid from sources enriched in ribonuclease. *Biochemistry*, **18**, 5294.

CHRONIC LEUKEMIA-MYELOMA TASK FORCE, NATIONAL CANCER INSTITUTE (1973). Proposed guidelines for protocol studies II. Plasma cell myeloma. *Cancer Chemotherapy Report*, **4**, 145.

DALTON, W.S., GROGAN, T.M., MELTZER, P.S. & 6 others (1989). Drug-resistance in multiple myeloma and non-Hodgkin's lymphoma: detection of P-glycoprotein and potential circumvention by addition of verapamil to chemotherapy. *J. Clin. Oncol.*, **7**, 415.

DULIK, D.M., FEUSELAU, C. & HILTON, J. (1986). Characterization of melphalan-glutathione adducts whose formation is catalysed by glutathione transferase. *Biochem. Pharmacol.*, **35**, 3405.

DURIE, B.G.M., DIXON, D.O., CARTER, S. & 7 others (1986). Improved survival duration with combination chemotherapy induction for multiple myeloma: a southwest oncology group study. *J. Clin. Oncol.*, **4**, 1227.

EPSTEIN, J., XIAO, H. & OBA, B.K. (1989). P-glycoprotein expression in plasma-cell myeloma is associated with resistance to VAD. *Blood*, **74**, 913.

FOJO, A., AKIYAMA, S.-I., GOTTESMAN, M.M. & PASTAN, I. (1985). Reduced drug accumulation in multiply-resistant human tumor KB carcinoma cell lines. *Cancer Res.*, **45**, 3002.

FOJO, A.T., UEDA, K., SLAMON, D.J., POPLACK, D.G., GOTTESMAN, M.M. & PASTAN, I. (1987). Expression of a multidrug-resistance gene in tumor and tissues. *Proc. Natl Acad. Sci. USA*, **84**, 265.

GROS, P., CROOP, J., RONINSON, I., VARSHAVSKY, A. & HOUSMAN, D.E. (1986). Isolation and characterization of DNA sequences amplified in multidrug-resistant hamster cells. *Proc. Natl Acad. Sci. USA*, **83**, 337.

ITO, Y., TANIMOTO, M., KUMAZAWA, T. & 4 others (1989). Increased p-glycoprotein expression and multidrug-resistant gene (mdr1) amplification are infrequently found in fresh acute leukemia cells. *Cancer*, **63**, 1534.

JOSHUA, D.E., SNOWDON, L., GIBSON, J. & 9 others (1991). Multiple myeloma: plateau phase revisited. *Haemat. Rev.*, **5**, 59.

KARTNER, N., RIORDAN, J. & LING, V. (1983). Cell surface P-glycoprotein associated with multidrug-resistance in mammalian cell lines. *Science*, **221**, 1285.

MEISTER, A. (1988). Glutathione metabolism and its selective modification. *J. Biol. Chem.*, **263**, 17205.

SCANLON, K.J., KASHANI-SABET, M., MIYACHI, H., SOWERS, L.C. & ROSSI, J. (1989). Molecular basis of cisplatin resistance in human carcinomas: model systems and patients. *Anticancer Res.*, **9**, 1301.

STOW, M.W. & WARR, J.R. (1991). Amplification and expression of the *mdr* genes and flanking sequences in verapamil hypersensitive hamster cell lines. *Biochim. Biophys. Acta*, **1092**, 7.

THIEBAUT, F., TSURUO, T., HAMADA, H., GOTTESMAN, M.M., PASTAN, J. & WILLINGHAM, M.C. (1987). Cellular localisation of the multidrug-resistance gene product P-glycoprotein in normal human tissues. *Proc. Natl Acad. Sci. USA*, **84**, 7735.

THORGEIRSSON, S.S., HUBER, B.E., SORRELL, S., FOJO, A., PASTAN, I. & GOTTESMAN, M.M. (1987). Expression of the multidrug resistant gene in hepatocarcinogenesis and regenerating rat liver. *Science*, **236**, 1120.

WOODCOCK, D.M., JEFFERSON, S., LINSENMEYER, M.E. & 4 others (1990). Reversal of the multidrug resistance phenotype with Cremophor EL, a common vehicle for water-insoluable vitamins and drugs. *Cancer Res.*, **50**, 4199.